# An 18.8–33.9 GHz, 2.26 mW Current-Reuse Injection-Locked Frequency Divider for Radar Sensor Applications

**DOI:** 10.3390/s21072551

**Published:** 2021-04-06

**Authors:** Kwang-Il Oh, Goo-Han Ko, Jeong-Geun Kim, Donghyun Baek

**Affiliations:** 1 Microwave Embedded Circuit & System (MECAS) Lab., School of Electrical Engineering, Chung-Ang University, 84 Heukseok, Dongjack, Seoul 06974, Korea; dhrhkddlf123@cau.ac.kr (K.-I.O.); rngks79@cau.ac.kr (G.-H.K.); 2 Integrated Radar Lab., Department of Electronic Engineering, KwangWoon University, 20 Gwangun, Nowon, Seoul 01897, Korea; junggun@kw.ac.kr

**Keywords:** current-reuse, injection-locked frequency divider, radar sensor, wideband

## Abstract

An 18.8–33.9 GHz, 2.26 mW current-reuse (CR) injection-locked frequency divider (ILFD) for radar sensor applications is presented in this paper. A fourth-order resonator is designed using a transformer with a distributed inductor for wideband operating of the ILFD. The CR core is employed to reduce the power consumption compared to conventional cross-coupled pair ILFDs. The targeted input center frequency is 24 GHz for radar application. The self-oscillated frequency of the proposed CR-ILFD is 14.08 GHz. The input frequency locking range is from 18.8 to 33.8 GHz (57%) at an injection power of 0 dBm without a capacitor bank or varactors. The proposed CR-ILFD consumes 2.26 mW of power from a 1 V supply voltage. The entire die size is 0.75 mm × 0.45 mm. This CR-ILFD is implemented in a 65 nm complementary metal-oxide semiconductor (CMOS) technology.

## 1. Introduction

Recently, the demand for radar sensors has been rapidly increasing with the development of the Internet of Things (IoT) industry and the autonomous vehicle industry. The complementary metal-oxide semiconductor (CMOS) radar is characterized by various operating methods such as doppler, frequency-modulated continuous wave (FMCW), and (continuous-wave) CW. In the doppler radar, a low frequency to millimeter-wave (mm-Wave) must be used to acquire a two-dimensional image through synthetic aperture radar (SAR). Bandwidths of 500 MHz or more are used to obtain high-resolution images [1,2]. In addition, wideband performance is very important in frequency-modulated continuous wave (FMCW) radars because wideband chirp is directly related to the high-resolution distance information [3]. Therefore, the wideband performance of the signal generator, the core of the sensor, is required [4,5].

**Figure 1:**
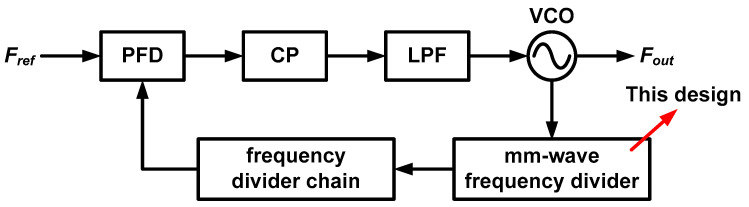
Conventional phase-locked loop with mm-Wave frequency divider.

Generally, the performance of the phase-locked loop (PLL) in the signal generator must be concerned to obtain low noise mm-Wave signals. Figure 1 shows the block diagram of conventional PLL structure that consists of a phase-frequency detector (PFD), charge pump (CP), low-pass filter (LPF), voltage-controlled oscillator (VCO) and frequency divider. The key blocks that determine the specification of the PLL in the mm-Wave band are VCO [6,7] and frequency divider [8]. The mm-Wave frequency divider should operate at high speed and should have a wide operating range for applying the wideband sensor applications.

Frequency dividers are designed as the current mode logic (CML) divider, regenerative divider, and LC oscillator-based injection-locked frequency divider (ILFD). The CML divider is a combination of two flip-flops that perform simple logical operations [9,10,11,12]. Generally, CML dividers have a wide operating range and occupy a small chip area with no inductor design. However, CML dividers suffer from large power consumption, limited maximum operation frequency, and process, voltage, and temperature (PVT) variation at the mm-Wave. To address these shortcomings, tunable self-resonant circuit [9], dynamic latches with load modulation [10,11], and additional calibration circuits [12] have been studied. However, these still consume large powers of 4.8 [11], and 6.2 mW [12], respectively. The regenerative divider and ILFD are also popular frequency dividers. These two types of frequency dividers are LC oscillator-based circuits and both of them are quite similar. The regenerative divider comprises an LC-based band pass filter (BPF) and active-type mixer [13,14,15]. The active type of mixer consumes power and takes over the role of -g_m_ core. Conversely, the ILFD comprises an LC-based BPF, -g_m_ core, and passive-type mixer that does not consume power. Therefore, regenerative dividers consume more power than ILFDs and are not generally used for mm-Wave applications because of the influence of many parasitic capacitors of the active-type mixer such as the Gilbert cell. The even-harmonic mixer [14] and digital-assisted circuit [15] are employed to widen the locking ranges of the regenerative divider. Their locking ranges are 33% and 57.4%, respectively. However, the highest input frequencies are limited to 18.4 and 14.8 GHz, consuming 10.8 and 12 mW power, respectively.

The most attractive mm-Wave frequency divider is the LC oscillator-based ILFD. The reasons for its high popularity are as follows. First, the ILFD self-oscillates when there is no input signal applied. It is possible to obtain a large output signal with a small input signal using the oscillator-based operation. Second, because of the LC resonator, the ILFD is advantageous for operation at the mm-Wave band. Finally, because the ILFD uses a passive type of mixer, it consumes less power than regenerative and CML dividers. However, the disadvantage is that the locking range is narrow because of a high-quality factor (Q) LC resonator. Several studies are being conducted to widen the locking range of ILFD [16,17,18]. The forward-body-bias techniques [16,17] are some of the effective ways of increasing the gain of the mixer and extending the locking range. Although the ILFD with the forward-body-bias techniques have a wide locking range of 90% in [17], there are several reasons why this technique is impractical in mm-Wave synthesizers. First, if a positive bias is applied to the body of an n-channel metal-oxide-semiconductor field-effect transistor (MOSFET), the leakage current cannot be ignored, and the possibility of a large diffusion current flow because of forward-bias increases. Second, the power of the harmonic signal increases because of non-linearity in devices. Applying an injection signal with an edge frequency in the locked range can make it difficult to distinguish the power difference between the output and harmonic signals. Finally, an additional circuit may be required to control the harmonic power, which can increase the circuit complexity and power consumption. The dual-resonance resonator is also considered as a suitable technique [18]. This ILFD has a locking range of 71.46%; however, it requires external bias control and has a small output power. Moreover, when a −3 dBm injection power is applied, an unlocking part occurs in the locking range.

In this paper, a low power and wide locking range LC oscillator-based current-reuse (CR) ILFD using a fourth-order resonator with the distributed inductor is proposed. The CR technique is employed to reduce power consumption. This paper is organized as follows. Section 2 presents an analysis of the ILFD locking range. The limitations of the maximum locking range and harmonic issues are also presented. Section 3 presents the circuit design of the proposed CR-ILFD including the modeling of the transformer and design flow chart. The measurement results are shown in Section 4. Finally, conclusions are organized in Section 5.

## 2. Locking Range Analysis of ILFD

**Figure 2:**
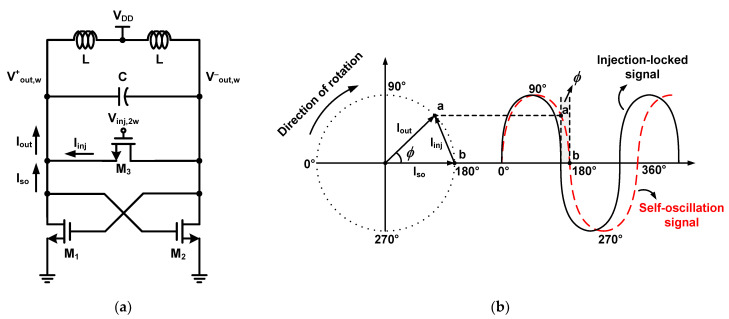
(**a**) Schematic of the conventional cross-coupled pair ILFD with second-order resonator and (**b**) phasor diagram for the basic principle of the conventional ILFD.

Figure 2a shows a schematic of the conventional cross-coupled pair ILFD with a second-order resonator. This ILFD consists of an N-channel metal-oxide semiconductor (NMOS) cross-coupled pair (M_1_, M_2_), injection switch (M_3_) and LC resonator. The ILFD self-oscillates if there is no injection signal at the gate of M_3_. Biasing the injection signal of V_inj,2w_ at the gate of M_3_, the ILFD outputs V^+^_out,w_ and V^−^_out,w_. When the frequency of the output signal is exactly half the frequency of the injection signal, it is referred to as “locking”. To easily understand the locking operation, the current is classified into three types, namely, *I_so_*, *I_inj_*, and *I_out_*. *I_so_* represents the self-oscillation current flowing through the core when the ILFD self-oscillates without an injection signal. *I_inj_* is the injection current flowing through M_3_ when an injection signal is applied. *I_out_* is the output current, which is the sum of *I_so_* and *I_inj_*. Figure 2b shows the phasor diagram for the three current types. The phasor rotates clockwise. Point “a” shows that the phase has changed from *I_so_* by *ϕ*. Point “b” shows the phase when the ILFD self-oscillates without an injection signal. The relational expression of the current vectors is as follows.

(1) $$I_{out}=I_{so}+I_{inj}.$$

Two waves are shown in Figure 2b, one is the self-oscillation signal of the ILFD and the other is the injection-locked signal. Point “b” of the self-oscillation signal is moved to point “a” by the injection signal. Therefore, the phase at 180° of the injection-locked signal is point “a” of the self-oscillation signal. Injection is instantaneously performed every half period, and the range of *ϕ* can be derived using the following equations.

(2) $$\phi =∠I_{out}=∠\left(I_{so}+I_{inj}\right),$$

(3) $$V_{out}=Z_{L}\cdot I_{out},$$

(4) $$∠I_{out}=∠V_{out}-∠Z_{L},$$

where *V_out_* is the output voltage signal when the ILFD is locked, and Z_L_ represents the load impedance of the LC resonator. Equation (4) can be derived using the phasor in (3). To replace *V_out_* with the self-oscillation and injection signals, the following equations are derived as

(5) $$V_{out}=V_{so}+V_{inj},$$

where *V_so_* is the output voltage signal when the ILFD self-oscillates and *V_inj_* is the injection voltage signal generated from M_3_. It should be noted that *V_inj_* is different from the input voltage signal, *V_inj,_*_2*w*_. According to Equations (4) and (5), the *ϕ* is calculated as

(6) $$\phi =∠\left(V_{so}\pm V_{inj}\right)-∠Z_{L}.$$

The sign of *V_inj_* is determined based on the value of the locked frequency relative to the self-oscillation frequency. When the ILFD self-oscillates with no injection signal, (6) is calculated as follows.

(7) $${\phi |}_{V_{inj}=0}=∠\;V_{so}-∠\;Z_{L}.$$

*V_inj_* is zero, and *V_so_* is expressed as the product of I_so_ and Z_L_. Because Z_L_ is canceled out, the following equation is satisfied:

(8) $${\phi |}_{V_{inj}=0}=∠I_{so}.$$

Meanwhile, *ϕ_max_* is derived when the following condition is satisfied:

(9) $$I_{out}⊥I_{inj}.$$

The largest angle between *I_so_* and *I_out_* can be realized by considering the phasor as shown in Figure 2b. This is the condition of (9) where *I_out_* and *I_inj_* are vertical. Using the trigonometric function,

(10) $$\sin\phi_{\max}=\pm \frac{\left|I_{inj}\right|}{\left|I_{so}\right|},$$

(11) $$\phi_{\max}=\pm \arcsin\left(\frac{\left|g_{inj}\cdot V_{inj}\right|}{\left|g_{m}\cdot V_{so}\right|}\right),$$

where *g_m_* and *g_inj_* represent the transconductance of the cross-coupled pair and injection switch, respectively.

According to (6), the conditions for extending the locking range of the ILFD can be determined qualitatively. First, the magnitude of the self-oscillation signal *V_so_* is decreased by reducing the sizes of M_1_ and M_2_ to decrease the transconductance of the cross-coupled pair. However, when the transconductance of the cross-coupled pair is too small, it can make failure in the self-oscillation, causing the ILFD to act as a harmonic buffer. Second, to increase the amplitude of *V_inj_* generated by M_3_, the size of M_3_ can be increased or the injection signal *V_inj,_*_2*w*_ can be amplified. However, the operation frequency may be limited by large parasitic capacitors. A pre-buffer, which consumes additional power, will be required to increase the amplitude of *V_inj,_*_2*w*_. Finally, the phase of the load impedance can be changed. The phase of Z_L_ can increase or decrease *ϕ*. However, the maximum and minimum values of the phase, ±*ϕ*_max_, limit the range of *ϕ*. Therefore, the phase of Z_L_ should be close to zero value in the wide frequency range. In conclusion, the maximum and minimum values of *ϕ* are determined by (11), and the method of extending the range of *ϕ* is consistent with the equation in (6).

**Figure 3:**
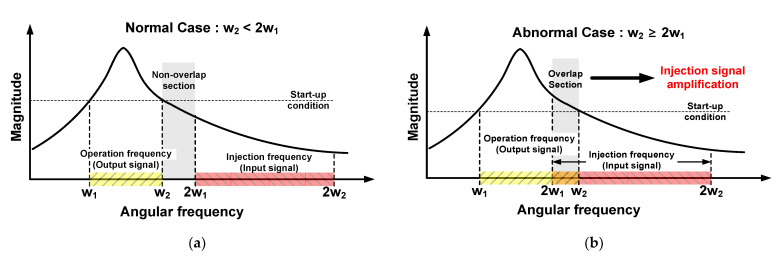
Graphs of magnitude of load impedance against angular frequency; (**a**) normal case, (**b**) abnormal case.

The power of the output signal should be greater than that of the input signal. Two graphs of the load impedance magnitude against the angular frequency are shown in Figure 3, which presents two cases. The first case is the normal case where the power of the input signal is significantly smaller than that of the output signal as shown in Figure 3a. The range from *w*_1_ to *w*_2_ is the operation frequency band obtained by dividing by two, and the range from 2*w*_1_ to 2*w*_2_ is the injection frequency band. The operation and injection frequency bands do not overlap in the normal case because 2*w*_1_ is larger than w_2_. Therefore, the input signal does not exceed the start-up condition and is not amplified more than the output signal. The second case is the abnormal case where the power of the input signal can be larger than that of the output signal, as in Figure 3b. Here, the operation and injection frequency bands overlap because 2*w*_1_ is smaller than *w*_2_. The injection frequency band contains the parts that exceed the start-up conditions, which are determined by the following “Barkhausen formula”.

(12) $$g_{m}\cdot \left|Z_{L}\right|\geq 1.$$

In the abnormal case, the ILFD cannot be used in mm-Wave applications, because the input and output signals are amplified together in the frequency band used.

This problem can be solved by increasing the division ratio of the ILFD. However, to operate at high division ratio, a harmonic signal with a small magnitude should be used, which results in a narrow locking range of the ILFD [19,20]. Additionally, the injection mixer for the high division ratio creates larger parasitic capacitance than the injection switch of the divide-by-two ILFD. Consequently, an ILFD that operates at a high division ratio greater than two is disadvantageous for application in the mm-Wave band. Therefore, a divide-by-two ILFD optimized to have a wide locking range without including the abnormal case would be most suited as a mm-Wave frequency divider. The following equation is used to calculate the locking range of the ILFD.

(13) $$LR=\frac{w_{2}-w_{1}}{w_{1}+\left(w_{2}-w_{1}\right)/2}\cdot 100\;\left(\%\right).$$

Under the normal case condition, *w*_2_ < 2*w*_1_, the maximum locking range of the divide-by-two ILFD can be obtained when w_2_ is equal to 2*w*_1_. Therefore, the maximum locking range is

(14) $${LR_{\max}|}_{w_{2}=2w_{1}}=66.7\%,$$

where *LR* is the locking range. If the locking range of the divide-by-two ILFD exceeds 66.7%, the power of the input signal may be greater than that of the output signal. In conclusion, the locking range of the ILFD should be designed to be less than 66.7%.

## 3. Circuit Design of Proposed CR-ILFD

### 3.1. Fourth-Order Resonator and CR Core

**Figure 4:**
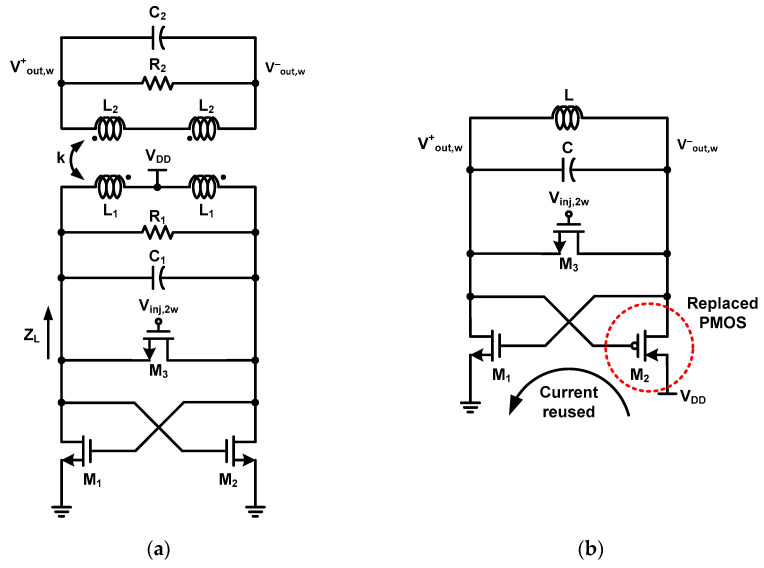
Schematic of (**a**) conventional cross-coupled pair ILFD with fourth-order resonator and (**b**) CR core-based ILFD.

As mentioned in the previous section, to extend the locking range of the ILFD, the phase plot of the load impedance should be flat in the range of ±*ϕ*_max_ [8,21]. A fourth-order resonator with two poles is required to flatten the phase plot. Figure 4a shows a schematic of the conventional cross-coupled pair-based ILFD with a fourth-order resonator consisting of a resonator (*L*_1_, *C*_1_, *R*_1_, *L*_2_, *C*_2_, *R*_2_), cross-coupled pair (M_1_, M_2_) and injection switch (M_3_). The “k” is the coupling factor between *L*_1_ and *L*_2_. *Z_L_* is the load impedance of the resonator, which is calculated as

(15) $$Z_{L}=\frac{(1-k^{2})L_{1}L_{2}C_{2}s^{3}+L_{1}s}{(1-k^{2})L_{1}L_{2}C_{1}C_{2}s^{4}+\left(L_{1}C_{1}+L_{2}C_{2}\right)s^{2}+1}.$$

*R*_1_ and *R*_2_ are resistors that affect the quality (Q) factor of the resonator and have been approximated in this calculation. Two poles that make the denominator zero are represented using the following equation [22],

(16) $$w_{R,L}=\sqrt{\frac{L_{1}C_{1}+L_{2}C_{2}\pm \sqrt{{\left(L_{1}C_{1}+L_{2}C_{2}\right)}^{2}-4(1-k^{2})L_{1}L_{2}C_{1}C_{2}}}{2(1-k^{2})L_{1}L_{2}C_{1}C_{2}}}.$$

Assuming that *L*_1_ = *L*_2_ and *C*_1_ = *C*_2_,

(17) $$w_{L,R}=\frac{1}{\sqrt{(1\pm k)LC}}.$$

According to (17), the distance between the two poles increases as the value of k increases and the distance between the two poles decreases as the k value decreases. If k is zero, the pole value is obviously equal to that of the second-order resonator (18). Figure 4b shows a schematic of the conventional ILFD with the CR core. For the CR core, M_2_ of the cross-coupled pair ILFD in Figure 2a is replaced by P-channel metal-oxide semiconductor (PMOS) [23,24,25,26,27]. The oscillation of the CR core can be divided into two half periods. In the first half period, the current flows through M_1_ and M_2_, and in the second half period, no current flows through M_1_ and M_2_. Unlike the oscillation in the cross-coupled pair core, the oscillation of the CR core reduces the current by simultaneously turning the MOSFET on and off [24].

**Figure 5:**
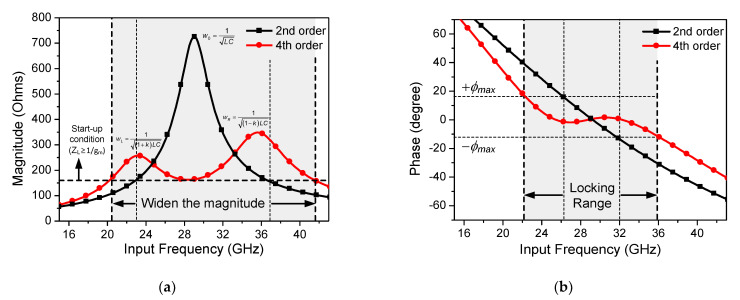
(**a**) Simulated magnitude plot and (**b**) phase plot of second-order resonator-based ILFD and fourth-order resonator-based ILFD.

Figure 5 shows the magnitude and phase plots of the second- and fourth-order resonator-based ILFDs. The schematic of the second-order resonator-based ILFD is shown in Figure 2a. Figure 5a shows the graph of the load impedance magnitude against the input frequency. The second-order resonator-based ILFD has one pole, w_0_, that is expressed as follows.

(18) $$w_{0}=\frac{1}{\sqrt{LC}}.$$

If the fourth-order rather than the second-order resonator-based ILFD is applied, the magnitude plot of the load impedance becomes wider even if the maximum magnitude value decreases. However, because a new minimum value occurs between the two poles, it is necessary to simulate whether locking is sufficiently achieved at this value. If the minimum value between the two poles is less than the start-up condition (12), the ILFD does not operate in that frequency range. Figure 5b shows the phase plot against the input frequency. According to (11), the ±*ϕ*_max_ limits the locking range of the ILFD. Unlike the phase of the second-order resonator-based ILFD, that of the fourth-order resonator-based ILFD has a value approximately equal to zero over a wide frequency range because of the formation of a ripple. Consequently, the simulated locking range of the ILFD is increased by 22% from 26–32 GHz (21%) to 22–36 GHz (43%).

### 3.2. Proposed CR-ILFD

**Figure 6:**
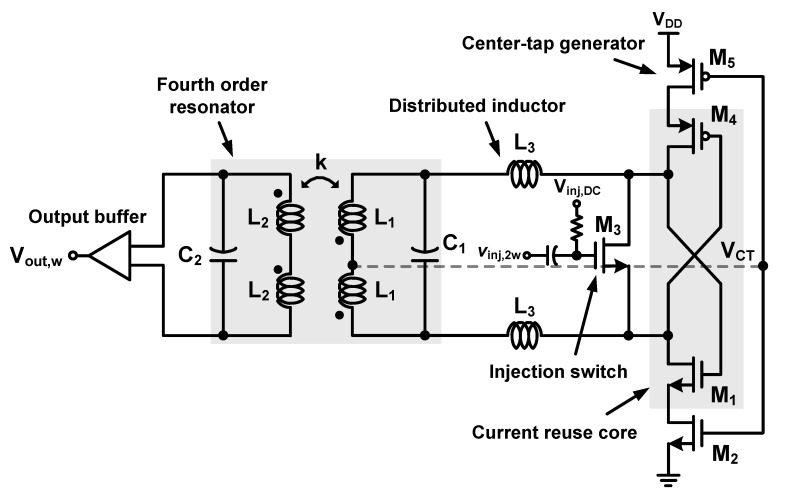
Schematic of the proposed CR-ILFD.

Figure 6 shows a schematic of the proposed CR-ILFD, consisting of a fourth-order resonator (*L*_1_, *C*_1_, *L*_2_, *C*_2_), distributed inductor (*L*_3_), injection switch (M_3_), CR core (M_1_, M_4_), center-tap generator (M_2_, M_5_), and output buffer. *V_inj,DC_* and *V_inj,_*_2*w*_ are the input signals, whereas *V_out,w_* is the output signal. The center-tap generator biases the node of the primary coil, *L*_1_ to *V_CT_*. If the g_m_ matching of PMOS and NMOS is well adjusted, mathematically, *V_CT_* would be *V_DD_*/2. The DC value of the injection switch can be biased to *V_CT_* without additional supply, but it was not connected for measurement. The distributed inductor is employed to extend the locking range of the ILFD. The distributed inductor is also referred to as the inductor distributed technique [28,29]. The magnitude of the load impedance can be increased by distributing the primary inductor into two series inductors.

**Figure 7:**
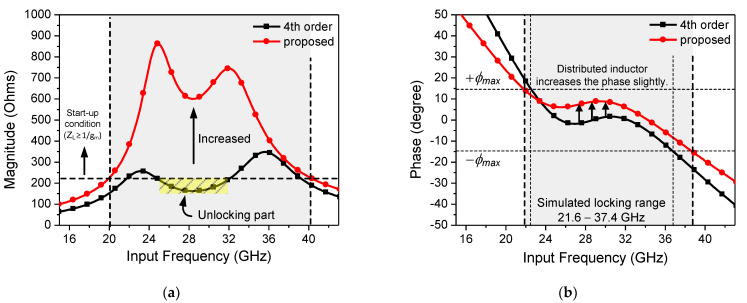
(**a**) Simulated magnitude plot and (**b**) phase plot of the fourth-order resonator-based ILFD and proposed CR-ILFD.

Figure 7a shows the simulated magnitude plot and phase plot of the fourth-order resonator-based ILFD and the proposed CR-ILFD with the fourth-order resonator with a distributed inductor. The start-up condition in Figure 7a is determined by the “Barkhausen formula” in (12). In the case of the fourth-order resonator-based ILFD, an unlocking part may occur because of the minimum value that is less than the start-up condition. However, the magnitude of the load impedance is sufficiently increased by using the inductor distributed technique. Figure 7b shows the slightly increased phase. This is not a critical amount of change because the phase ripple still exists between the ±*ϕ*_max_. The simulated locking range of the proposed ILFD is from 21.6 to 37.4 GHz, which is limited by the ±*ϕ*_max_ in (11).

**Figure 8:**
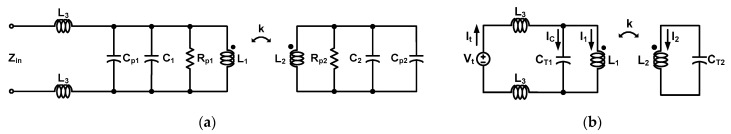
(**a**) Modeling of the fourth-order resonator using a transformer with distributed inductor. (**a**) Modeling of including the parasitic capacitors and resistors. (**b**) Approximate modeling applied to simplify calculations.

Figure 8 shows an equivalent model of the fourth-order resonator using a transformer with the distributed inductor. Figure 8a shows a model including the parasitic capacitors and resistors of the passive components. Zin is the input impedance and “*k*” is the coupling factor between *L*_1_ and *L*_2_. *C_p_*_1_, *R_p_*_1_, *C_p_*_2_, and *R_p_*_2_ represent the parasitic components. In the mm-Wave band, the analog circuits are affected more by electromagnetism. Therefore, the modeling of the resonator must be considered at the initial design stage. Because analyzing every parasitic component is difficult, modeling should be simplified by approximation as shown in Figure 8b. *C_T_*_1_ is the sum of *C_p_*_1_ and *C*_1_. Similarly, *C_T_*_2_ is the sum of *C_p_*_2_ and *C*_2_. Additionally, the Q factor of the inductor includes the parasitic resistances. *V_t_* and I_t_ are the test voltage and test current, respectively. *V_t_*/*I_t_* is equal to *Z_in_* in the simplified model. The value of *Z_in_* is calculated as follows.

(19) $$Z_{in}(s)=\frac{(1-k^{2})L_{1}L_{2}C_{T2}s^{3}+L_{1}s}{(1-k^{2})L_{1}L_{2}C_{T1}C_{T2}s^{4}+\left(L_{1}C_{T1}+L_{2}C_{T2}\right)s^{2}+1}\times (1+2L_{3}C_{T1}s^{2}).$$

If the distributed inductor (*L*_3_) is zero, then (19) is equal to (15). That is, the distributed inductor does not directly affect the pole value in (17), and if the distributed inductor value is increased, the magnitude of *Z_in_* can be increased.

The design parameters are listed in Table 1. Because the center-tap generator should not limit the core operation, the width of the center-tap generator should be significantly larger than that of the CR core. The parasitic capacitor of the center-tap generator is separated from the resonator and does not affect the operating frequency. The sizes of the CR core and injection switch are not only determined by (11) and (12), but also by the influence of the parasitic capacitors.

**Figure 9:**
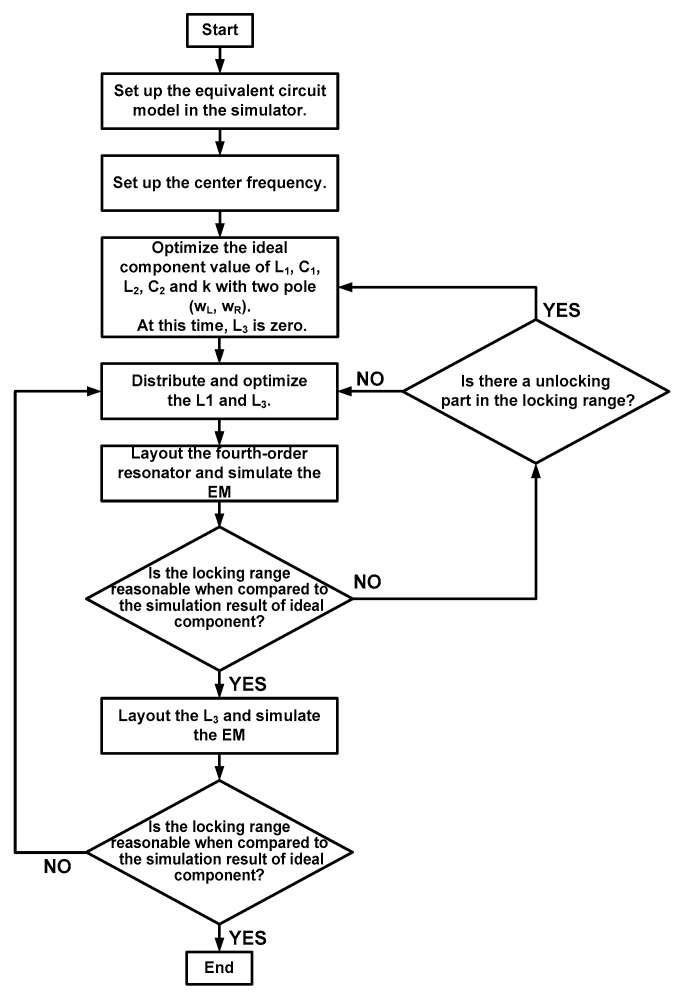
Flowchart of the design approach for the proposed CR-ILFD.

Figure 9 shows a flowchart of the design approach for the proposed CR-ILFD. First, the equivalent circuit model must be implemented in the simulator. Second, the values of the design parameters should be determined. In the proposed CR-ILFD, the center frequency is set to receive an injection signal of 28 GHz. Because the distributed inductor does not directly affect the pole value, the values of *L*_1_, *C*_1_, *L*_2_, *C*_2_ and k are first determined. Subsequently, *L*_1_ is divided into two series inductors, *L*_1_ and *L*_3_. In this design, *L*_1_, *L*_2_, and *L*_3_ are 230, 265, and 433 pH, respectively. *C*_1_ and *C*_2_ are 144 and 240 fF, respectively. The value of k is 0.51. When *k* < 0.5, which represents a weak coupling, the distance between the poles increases, and a wide magnitude plot of the load impedance can be obtained. However, a coupling that is too weak can cause an unlocking part in which the ILFD does not work. Considering the locking range and unlocking part, the proposed CR-ILFD is designed with a coupling factor of 0.51. Finally, the layout and locking simulation are repeated in the order shown in the flowchart. Electromagnetic simulation is essential in the mm-Wave band. Therefore, it should be ensured that the difference between the equivalent modeling and implementation in the simulation of this circuit is reasonable.

## 4. Measurement Results

**Figure 10:**
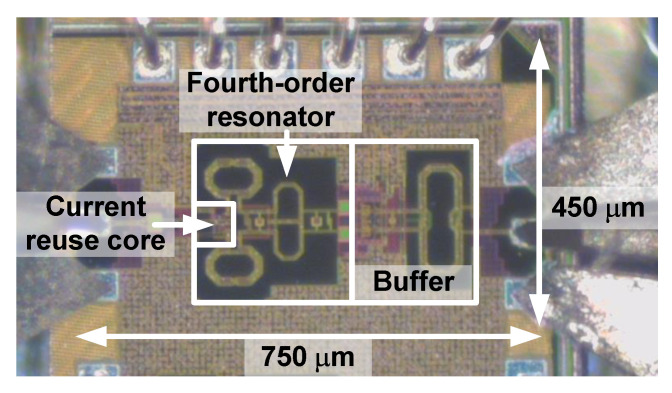
Die photograph of the proposed CR-ILFD.

**Figure 11:**
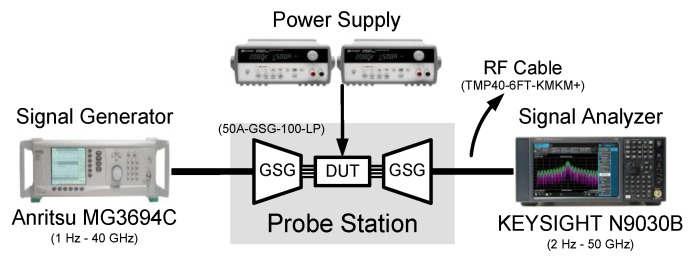
Measurement setup for the proposed CR-ILFD.

Figure 10 shows the die photograph of the proposed CR-ILFD, which was fabricated in a 65 nm CMOS technology. The die size including the entire pad is 0.75 mm × 0.45 mm and the chip size including the core and output buffer is 0.49 mm × 0.3 mm. The measurement setup for the proposed CR-ILFD is shown in Figure 11. The measurements were obtained using a probe station. The DC voltage was biased from the power supply. The CR core of the proposed CR-ILFD consumes 2.26 mW from a 1 V supply voltage, when no signal is applied to the injection switch. As *V_inj,DC_* increases, the power consumption increases. When *V_inj,DC_* is 0.7 V, the power consumption of the core increases by approximately 0.5 mW. The power consumption of the output buffer is approximately 3 mW. The injection signal was generated by Anritsu MG3694, which can generate frequencies up to 40 GHz. The output signal of the proposed CR-ILFD is analyzed by KEYSIGHT N9030B, which can analyze frequencies up to 50 GHz. When conducting measurements using mm-Wave signals, several losses occur around the device under test (DUT). Therefore, the calibration tests must be carried out carefully. In this measurement, the ground–signal–ground (GSG) probe tip has a loss of approximately 2.5 dB and that of the radio frequency (RF) cable has approximately 3 dB. Approximately a 1 dB loss occurs even when the signal generator output is 10 dBm. The loss of the signal generator was analyzed by connecting the signal analyzer and RF cable. All losses described above are based on the 28 GHz signal. Generally, the loss increases as the frequency increases, and decreases as the frequency decreases.

**Figure 12:**
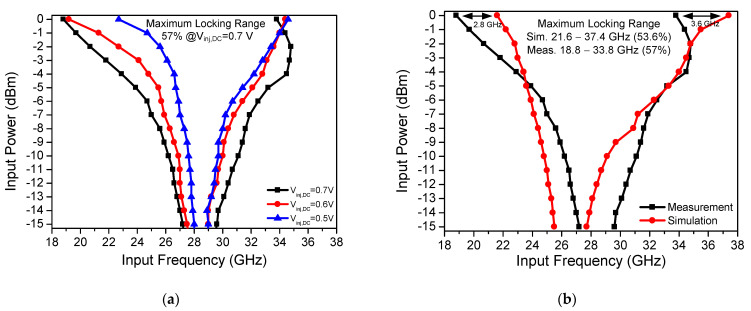
(**a**) Measured locking range results of the proposed CR-ILFD with different V_inj,DC_; (**b**) measured and simulated locking range results of the proposed CR-ILFD.

Figure 12a shows the measured locking range of the proposed CR-ILFD with different *V_inj,DC_* values. The maximum locking range is from 18.8 to 33.8 GHz (57%) at *V_inj,DC_* of 0.7 V. When the *V_inj,DC_* is biased to 0.6 V, the locking range is from 19.2 to 34.4 GHz (56.7%), and when the *V_inj,DC_* is biased to 0.5 V, the locking range is reduced from 22.7 to 34.6 GHz (41.5%). The above ranges were obtained from 0 dBm input power and 1 V supply voltage. As the *V_inj,DC_* decreases, the locking range also tends to decrease. Figure 12b shows a comparison of the measured and simulated locking range results of the proposed CR-ILFD. The measured locking range is 57%, and simulated locking range is from 21.6 to 37.4 GHz (53.6%). When 0 dBm input power is injected to the CR-ILFD, the measured locking range is typically changed to a lower frequency band than the simulated locking range. The operating frequency band was lowered by approximately 3 GHz. This is because of various electromagnetic components, such as RF pads, printed circuit board (PCB), and metal lines that were not considered in the simulations. The measured maximum operation frequency is higher when the input power is −3 dBm compared to when the input power is 0 dBm. This is because of the saturation of the input signal level.

**Figure 13:**
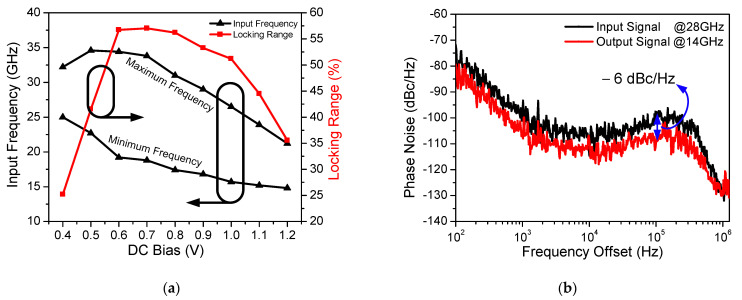
(**a**) Measured maximum and minimum operation frequency of the proposed CR-ILFD with different *V_inj,DC_*; (**b**) Measured phase noise of input and output signal.

Figure 13a shows the measured maximum and minimum operation frequencies of the proposed CR-ILFD with different *V_inj,DC_* values. This measurement was carried out with 0 dBm input power and 1 V supply voltage. *V_inj,DC_* is swept from 0.4 to 1.2 V, and the widest locking range is obtained at the *V_inj,DC_* of 0.7 V. When *V_inj,DC_* increases from 0.7 V, the maximum and minimum operation frequencies decrease, and the locking range also decreases.

**Figure 14:**
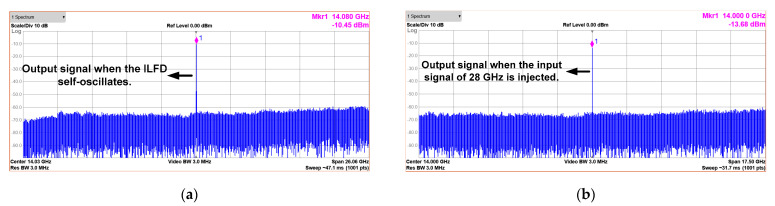
Spectrums of the output signal (**a**) when the proposed CR-ILFD self-oscillates; (**b**) when the proposed CR-ILFD is locked with a 28 GHz injection signal.

**Figure 15:**
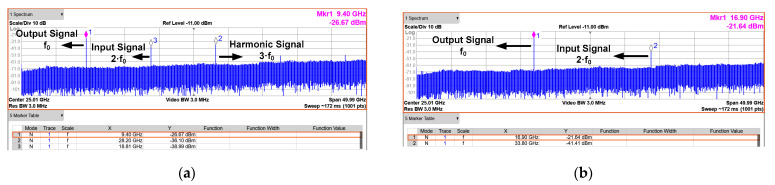
Full span spectrums (**a**) when the minimum input frequency is injected (18.8 GHz); (**b**) when the maximum input frequency is injected (33.8 GHz). The power difference between the output and input signals is approximately 10 dB or more.

The measured phase noise of the input and output signal is shown in Figure 13b. The 28 GHz input signal is generated by Anritsu MG3694, which is applied to the proposed CR-ILFD and the output signal is 14 GHz. The phase noise of the output signal is −109.57 and −129.81 dBc/Hz at 100 kHz and 1 MHz offset frequency, respectively. The phase noise of the output signal should be measured at 6 dBc/Hz lower than that of the input signal because the input signal frequency is twice that of the output signal. Figure 14 and Figure 15 show the results of several spectrums of the CR-ILFD’s output signal measured using the KEYSIGHT N9030B. The spectrum of the output signal when the proposed CR-ILFD self-oscillates is shown in Figure 14a. The output frequency is 14.08 GHz, and output power is −10.45 dBm. If the loss of the RF cable and GSG probe tip is calibrated, the output power will be approximately −5 dBm. The spectrum of the output signal when the 28 GHz input signal is injected to the proposed CR-ILFD is shown in Figure 14b. The frequency of the output signal is 14 GHz, which is exactly half the frequency of the input signal. The output power is approximately −8 dBm with loss calibration. Figure 15a shows the full span spectrum when the minimum input frequency, 18.8 GHz, is injected. Three tones are visible in the spectrum: the output signal (f_0_), input signal (2f_0_), and harmonic signal (3f_0_). As shown in Figure 3, several harmonic components are amplified at output when the minimum input frequency is injected to the CR-ILFD. Locking is possible even if a lower input frequency is injected. However, the input signal is amplified such that the power difference from the output signal is less than 10 dB. When 18.8 GHz is injected, the power difference between the desired output signal and the harmonic signal is approximately 10 dB. Figure 15b shows the full span spectrum when the maximum frequency input signal of 33.8 GHz is injected. The power difference between the output and input signals is more about 20 dB. It can be observed that the amplified input signal is smaller when the maximum input frequency is injected than when the minimum input frequency is injected. As a result, harmonic rejection ratio of the input signal over the entire locking range is more than 10 dBc.

Table 2 summarizes the performance comparison of different core ILFDs. These include challenging and typical ILFD cores such as Darlington [30], Armstrong [31], Collpits [32], and cross-coupled pair [33,34,35]. This work has the highest figure of merit (FOM) compared to other ILFDs presented in Table 2.

Table 3 summarizes the performance comparison of the mm-Wave ILFDs [36,37,38,39,40,41]. ILFDs with division ratio greater than two are also included such as four [37,38] and six [41], but still have the highest FOM_1_ values.

## 5. Conclusions

This paper presents the wide locking range and low-power divide-by-two CR-ILFD. The fourth-order resonator is applied to extend the narrow operating range of the ILFD. In addition, the CR core decreases the power consumption. The input frequency locking range is from 18.8 to 33.8 GHz (57%) at an injection power of 0 dBm. The full-span spectrums at the maximum or minimum frequency are presented. The power difference between the output and harmonic signals is approximately 10 dB or more over the entire locking range. The proposed CR-ILFD dissipates 2.26 mW from a 1 V supply voltage and the die size is 0.75 mm × 0.45 mm. This CR-ILFD is implemented in a 65 nm CMOS technology.

## Figures and Tables

**Table 1:** Design parameters of the proposed CR-ILFD.

Design Parameter	Value
M_1_, M_2_, M_4_, M_5_ (unit W/L)	2 μm/0.06 μm
M_3_ (unit W/L)	1 μm/0.06 μm
Finger of M_1_, M_3_, M_4_	20
Finger of M_2_, M_5_	50
L_1_	230 pH
L_2_	265 pH
L_3_	433 pH
k	0.51
C_1_	144 fF
C_2_	240 fF

**Table 2:** Performance comparison of different core ILFDs.

	**This Work**	[30] 15′MTT	[31] 09′MWCL	[32] 08′MWCL	[33] 14′MWCL	[34] 15′APMC	[35] 17′MWCL
Technology	65-nm CMOS	0.18-μm CMOS	0.18-μm CMOS	0.18-μm CMOS	0.18-μm SiGe BiCMOS	0.18-μm CMOS	0.18-μm CMOS
Core topology	Current reuse	Darlington	Armstrong	Colpitts + Current reuse	Complementary cross-coupled pair	NMOS cross-coupled pair	NMOS cross-coupled pair
Self-oscillation frequency (GHz)	14.08	N/A	4.77–5.08 (w/varactor)	5.85–6.17 (w/varactor)	N/A	N/A	2.97–4.66 (w/varactor)
Input signal power (dBm)	0	0	0	0	0	0	0
Division ratio	2	2	2	2	2	2	4
Input frequency range (GHz)	18.8–33.8	20.5–22.9	7.7–11.5	7.3–14.4	20.1–25.9	10.2–15.5	13–19
Locking range (%)	57	11	39.6	65.4	25.1	41.4	37.5 *
Supply voltage (V)	1	1.2	1.4	1.5	1.8	1.2	0.8
Power consumption of core (mW)	2.26	1.73	9.02	7.65	4.8	2.71	7.09
Phase noise (dBc/Hz @1 MHz)	−129.81 (14 GHz)	−138.3 (N/A)	−134.942 (4.9 GHz)	−134.8 (6 GHz)	−124 (12.5 GHz)	−120.53 (5.495 GHz) @ 100 kHz	−133.26 (4 GHz)
FOM_1_ (GHz/mW)	6.64	1.38	0.42	0.93	1.21	1.96	0.85
FOM_2_ (GHz/mW)	13.28	2.76	0.84	1.86	2.42	3.92	3.4
Chip size (mm^2^)	0.75 × 0.45	0.8 × 0.75	0.55 × 0.74	0.46 × 0.52	0.75 × 0.78	0.57 × 0.68	1.01 × 1.18

FOM_1_ = Input frequency range/power consumption [GHz/mW], FOM_2_ = (Input frequency range × division ratio)/power consumption [GHz/mW], *: Total locking range (low band + high band).

**Table 3:** Performance comparison of mm-Wave ILFDs.

	This Work	[36] 15′MWCL	[37] 13′TCAS1	[38] 11′MTT	[39] 17′JSSC	[40] 09′ISSCC	[41] 20′MWCL
Technology	65-nm CMOS	65-nm CMOS	65-nm CMOS	0.13-μm CMOS	0.13-μm CMOS	0.13-μm CMOS	90-nm CMOS
Self-oscillation frequency (GHz)	14.08	17.5	N/A	5.9	25.9	N/A	9.7
Input signal power (dBm)	0	0	0	0	0	0	−5
Division ratio	2	2	4	4	2	2	6
Input frequency range (GHz)	18.8–33.8	31.7–39.3	58.5–72.9	13.5–30.5	35–4441–59.5	35.6–39.3	54.5–60.1
Locking range (%)	57	21.4	21.9	77.3	53 *	9.9	9.8
Supply Voltage (V)	1	1	0.6	1.4	1.15	1	N/A
Power consumption of core (mW)	2.26	2.5	2.2	7.3	3.8	3.12	5.6
Phase noise (dBc/Hz @1 MHz)	–129.81 (14 GHz)	–102 (N/A)	–126.74 (N/A)	–137.4 (6 GHz)	–124 (24 GHz)	–133.7 (N/A)	–140 (9.7 GHz)
FOM_1_ (GHz/mW)	6.64	3.04	6.54	2.33	6.45	1.19	1
FOM_2_ (GHz/mW)	13.28	6.08	26.16	9.32	12.9	2.38	6
Chip size (mm^2^)	0.75 × 0.45	0.6 × 0.75	0.16 × 0.26	0.52 × 0.64	1 × 0.9	0.13 × 0.18 **	0.83 × 0.61

*: Total locking range (low band + high band), **: Only core size.

## Data Availability

No new data were created in this study. Data sharing is not applicable to this article.
